# Negative Perceptions of Aging and Decline in Walking Speed: A Self-Fulfilling Prophecy

**DOI:** 10.1371/journal.pone.0123260

**Published:** 2015-04-29

**Authors:** Deirdre A. Robertson, George M. Savva, Bellinda L. King-Kallimanis, Rose Anne Kenny

**Affiliations:** 1 TILDA (The Irish Longitudinal Study on Ageing), Department of Medical Gerontology, Trinity College, Dublin, Ireland; 2 School of Health Sciences, University of East Anglia, Norwich Research Park, Norwich, Norfolk, United Kingdom; 3 Trinity College Institute of Neuroscience, Trinity College, Dublin, Ireland; Cardiff University, UNITED KINGDOM

## Abstract

**Introduction:**

Walking speed is a meaningful marker of physical function in the aging population. While it is a primarily physical measure, experimental studies have shown that merely priming older adults with negative stereotypes about aging results in immediate declines in objective walking speed. What is not clear is whether this is a temporary experimental effect or whether negative aging stereotypes have detrimental effects on long term objective health. We sought to explore the association between baseline negative perceptions of aging in the general population and objective walking speed 2 years later.

**Method:**

4,803 participations were assessed over 2 waves of The Irish Longitudinal Study on Ageing (TILDA), a prospective, population representative study of adults aged 50+ in the Republic of Ireland. Wave 1 measures – which included the Aging Perceptions Questionnaire, walking speed and all covariates - were taken between 2009 and 2011. Wave 2 measures – which included a second measurement of walking speed and covariates - were collected 2 years later between March and December 2012. Walking speed was measured as the number of seconds to complete the Timed Up-And-Go (TUG) task. Participations with a history of stroke, Parkinson’s disease or an MMSE < 18 were excluded.

**Results:**

After full adjustment for all covariates (age, gender, level of education, disability, chronic conditions, medications, global cognition and baseline TUG) negative perceptions of aging at baseline were associated with slower TUG speed 2 years later (B=.03, 95% CI = .01 to 05, p< .05).

**Conclusions:**

Walking speed has previously been considered to be a consequence of physical decline but these results highlight the direct role of psychological state in predicting an objective aging outcome. Negative perceptions about aging are a potentially modifiable risk factor of some elements of physical decline in aging.

## Introduction

“The true evil of old age is not the weakening of the body, but the indifference of the soul”

André Maurois An Art of Living [1]

Walking speed is a simple but meaningful marker of adverse outcomes in aging. Previous work has shown that adults who experience a significant decline in walking speed over just 2 years have a 90% increased risk of mortality, while slow walking speed can also predict future hospitalisation and disability [2, 3]. Current models describe the association between walking speed and adverse outcomes in aging as a reflection of advancing sarcopenia, increased inflammatory processes or chronic disease leading to death [2, 4]. There is, however, an increasing body of research suggesting that how people think about aging can affect how they age.

Aging is an automatic category by which humans naturally define each other. Along with other automatic categories, such as race and gender, it is subject to group biases such that humans in one age, race or gender group define those in other groups more negatively than those in their own group. The negative beliefs about other groups are referred to as stereotypes. Unlike race and gender, however, age is not a stable category but rather humans are part of different age cohorts throughout life. How, so, do people reconcile the negative stereotypes elicited about older age groups when they are younger with their own experiences when they themselves become part of an older age cohort? Becca Levy proposes that these negative stereotypes do not change or disappear but become internalised such that people who held negative stereotypes about older people when they were younger hold those beliefs to be true to themselves as they age [5]. Negative stereotypes originally held about other people therefore become negative self-perceptions of aging in later life. Further work may explain the cognitive mechanism behind this as older adults who are either primed with negative stereotypes about aging, or who already hold implicit negative views of aging, show decreased self-perception, in the form of self-esteem, when reminded of the similarities between themselves and other older adults [6].

Unsurprisingly, a number of studies have illustrated the negative effects of holding negative perceptions of aging on factors of psychological well-being including life satisfaction and mood [6–8]. Interestingly, however, a growing body of research is indicating that negative aging perceptions may not only affect psychological well-being as people age but also physical health. A number of studies have shown that holding negative perceptions of aging at baseline are associated with a decrease in self-reported performance in activities of daily living (ADLs), increased number of illnesses, decreased self-reported physical function, self-rated health and increased risk of mortality [7–12]. Aside from mortality and illness, however, these outcomes are self-reported, subjective assessments of function which may be confounded by the subjective nature of perceptions of aging. One more recent study by Sargent-Cox et al. showed that older adults with negative perceptions of aging exhibited decline in a composite measure of objective physical function over 16 years [13].

We sought to expand upon the work of Sargent-Cox and colleagues by investigating whether specific aspects of perceptions of aging are associated with decline in a physical health measure. Perceptions of aging encompass a range of cognitions including emotional responses to aging, feelings of control, and expectations for health, behavioural or social changes. The studies to date used uni-dimensional measures of aging perceptions which encompass these cognitions within the scale but which cannot separate the components to examine their independent effects on health. We thus investigated the impact of perceptions of aging using the short form of the Aging Perceptions Questionnaire (B-APQ) which includes 5 domains: the saliency of aging in one’s thoughts, emotional reactions to aging, expectations for positive consequences, feelings of control and expectations for negative consequences.

In addition, we used a specific and simple measure of walking speed that it is commonly used in clinical settings. The Timed Up-And-Go task is a strong correlate of frailty which is a state of vulnerability to stressors and one of the biggest predictors of disability, hospitalisation and nursing home admission in older adults [14, 15]. We thus sought to investigate which of the five domains of aging perceptions encompassed within the B-APQ could predict longitudinal decline in performance on the TUG task in a population representative sample of older adults. We hypothesised that negative emotional responses to aging, feelings of lack of control and negative expectations would predict decline in performance while positive responses and feelings of control would be protective. We did not make a directional hypothesis for the saliency of aging related thoughts.

## Methods

### Sample

The Irish Longitudinal Study on Ageing (TILDA) is a large prospective cohort study of the social, economic and health circumstances of community-dwelling older people in Ireland. Analyses are based on the first wave of data collected between January 2009 and July 2011 and follow-up data collected between March and December 2012. The mean time difference between interviews was 744.12 days (24 months) (inter-quartile range = 114). The Irish Geodirectory—a listing of all residential addresses in the Republic of Ireland—was used as a sampling frame to randomly select households in which individuals aged 50+ lived. The full procedure has been described in detail elsewhere [16]. Household residents aged 50 or over and their spouses/partners (of any age) were eligible to participate in the study. The response rate was 62% leading to a final sample size of 8,504 participants. Ethical approval was obtained from the Trinity College Dublin Research Ethics Committee and all participants provided written informed consent.

The study design has been described in detail elsewhere [16]. Briefly, data collection involved (i) a computer-assisted personal interview carried out by trained social interviewers that included detailed questions on socio-demographics, wealth, health, lifestyle, social support and participation and use of health and social care (ii) a self-completion questionnaire which included questions about perceptions of aging, social networks and social participation (iii) a health assessment carried out by research nurses. Participants who were unwilling or unable to attend a health assessment in a designated health centre were offered a home-based health assessment. In wave 2 the computer-assisted personal interview and key elements of the health assessment were combined into a home interview and assessment carried out by trained interviewers.

**Fig 1:**
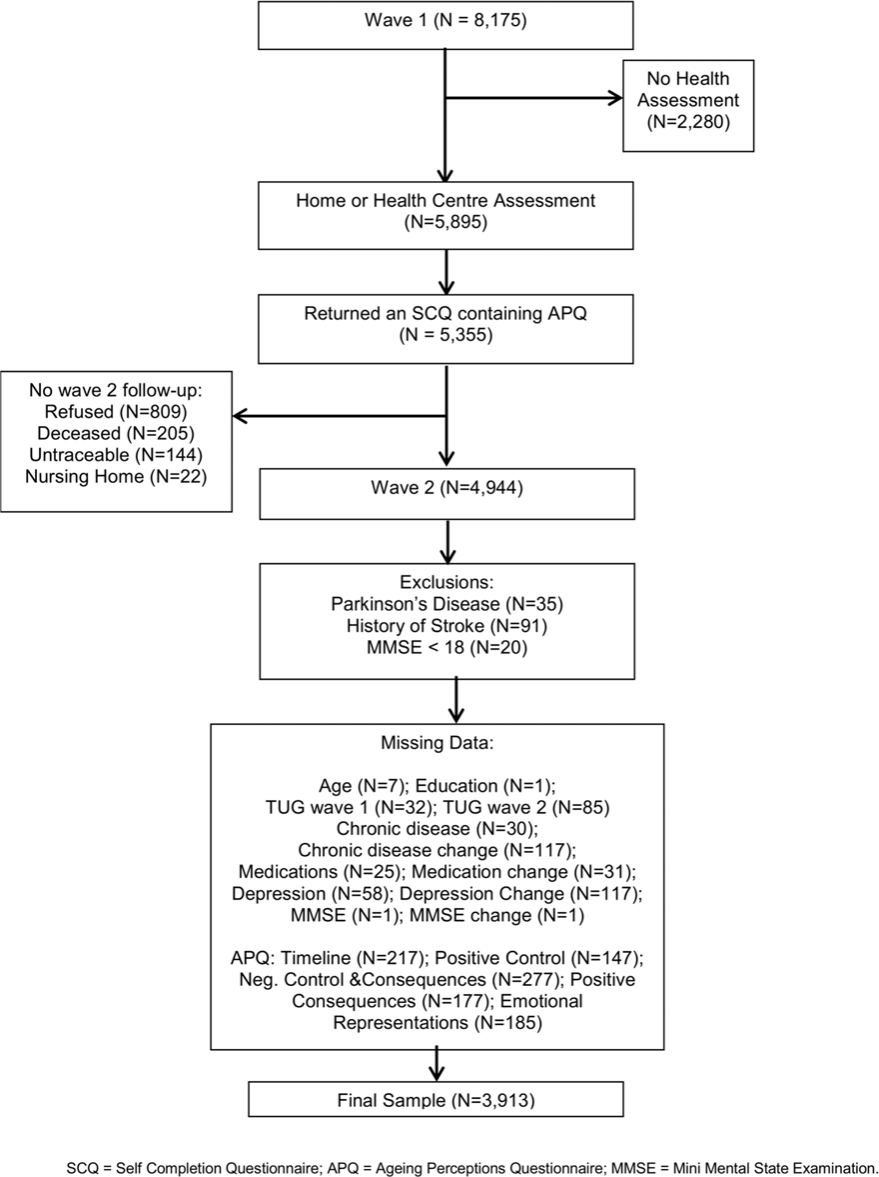
Flow Chart. Flow Chart Indicating Participants Remaining From Waves 1–2.

In total, 8,175 individuals aged 50 years and over were interviewed at baseline (N = 8,504 includes partners younger than 50) and 5,895 (72%) of these individuals also completed a health assessment. Follow up data for wave 2 was available for 6,995 (86%) participants of which 78 (.01%) were proxy interviews and were thus not included in the sample for this analysis. Follow up data was unavailable for 1,180 (14%) participants due to death (n = 205); refusal (n = 809) or no contact (n = 166). We also had a number of missing data values which are outlined in Table 1. Exclusion criteria for the present analysis included a doctor’s diagnosis of Parkinson’s disease (N = 35), a history of stroke (N = 91) or an MMSE of <18 (N = 20) at baseline or between waves 1 and 2. Our final sample included participants who had completed both a wave 1 health assessment and a wave 2 follow up, who had returned the self-completion questionnaire and who did not meet any of the exclusion criteria. The final sample size was 4,803 (for a flow chart of sample selection see Fig 1).

**Table 1:** Descriptive Statistics^a^ ^b^ ^a^ ^b^.

**Variable (Possible Range)**	**Total Sample**	**Dead**	**Refused/No Follow-Up**
	N = 4803	N = 205	N = 975
**Age**	62.8 (9.0)	73.9 (11.2)***	64.3 (10.2)***
	Missing = 7		
**Sex (female)**	54.6% (N = 2,624)	43.4% (N = 89)**	55.8% (N = 544)
	Missing = 0		
**Education (third level)**	35.1% (N = 1,685)	16.2% (N = 33)***	22.5% (N = 219)***
	Missing = 1		
**TUG time 1**	8.9 (3.0)	13.5 (7.1)***	9.5 (3.3)***
	Missing = 32		
**TUG time 2**	9.8 (3.9)	NA	NA
	Missing = 85		
**No. of Chronic Diseases**	1.4 (1.4)	2.0 (1.5)***	1.3 (1.4)
	Missing = 30		
**Disability**	Missing = 0		
No disability	90.03% (N = 479)		
IADL only	2.33% (N = 112)		
ADL only	5.00% (N = 240)		
IADL and ADL	2.64% (N = 127)		
**No. of Medications**	2.5 (2.6)	4.4 (3.3)***	2.4 (2.6)***
	Missing = 25		
**Depression (CES-D) (0–60)**	8.0 (6.3)	7.1 (7.4)**	6.2 (7.7)**
	Missing = 58		
**MMSE (0–30)**	28.5 (1.8)	26.1 (3.5)***	27.6 (2.8)***
	Missing = 1		
**Domains of APQ**			
**Timeline (0–5)**	2.4 (.8)	2.9 (1.1)***	2.5 (.87)*
	Missing = 217		
**Positive Control (0–5)**	4.0 (.6)	4.0 (.69)	3.8 (.77)***
	Missing = 147		
**Negative Control and Consequences (0–5)**	2.8 (.7)	3.4 (.85)***	3.0 (.80)***
	Missing = 277		
**Positive Consequences (0–5)**	3.8 (.7)	3.7 (.80)	3.7 (.72)***
	Missing = 177		
**Emotional (0–5)**	2.3 (.8)	2.4 (.87)	2.4 (.83)*
	Missing = 185		

Characteristics of the total sample compared to participants who died following wave 1 and those who refused follow up.

^a^***p <.001,

**p <.01,

* p <.05. Statistical significance was calculated using one way ANOVA for continuous variables and chi-square tests for categorical variables.

^b^TUG = Timed Up-and-Go task; CES-D = Centre for Epidemiological Studies Depression Scale; MMSE = Mini Mental State Examination; APQ = Aging Perceptions Questionnaire.

### Data Collection

#### Covariates

All covariates were measured at baseline and at wave 2. Longitudinal analyses controlled for change in covariates between waves.

Age, gender, employment and education were obtained by self-report. Education was categorised as having completed some or all of primary schooling, having completed some or all of secondary schooling or having completed any third level education or higher.

Global cognition was assessed using the Mini Mental State Examination (MMSE) [17]. Chronic conditions were ascertained by self-report of a doctor’s diagnosis and included: joint problems, cataracts, glaucoma, age-related macular degeneration, lung disease, asthma, arthritis, osteoporosis, cancer, Parkinson’s disease, peptic ulcer, liver disease, varicose ulcer, alcohol or substance abuse, chronic pain and incontinence. Number of chronic conditions were included in this analysis. Basic and instrumental activities of daily living (ADLs and IADLs) were collected through self-report. Participants were asked “Because of a health or memory problem, do you have any difficulty doing any of the activities on this card?” They were then shown a card listing 6 basic ADLs including dressing, walking across a room, bathing or showering, eating, getting in and out of bed and using the toilet. An ADL disability was defined as difficulty in at least one of the activities listed on the card. Participants were then asked the same question and given a second card listing instrumental ADLs (IADLs) including preparing a hot meal, doing household chores, shopping for groceries, making telephone calls, taking medications and managing money. An IADL disability was defined as difficulty in at least one of these tasks. The level of disability with each activity was not assessed. For cross-sectional analyses participants were classified into 4 categories: no disability, ADL only, IADL only or both ADL and IADL. For longitudinal analyses we assessed change in disability between waves. Due to the small numbers in groups when change in disability was categorised into both ADLs and IADLs we collated the information and assessed 4 levels: no disability in wave 1 or wave 2, stable level of disability in wave 2, reduced level of disability since wave 1, any new disability in wave 2. Participants were also asked to record all of the medications taken on a regular basis and interviewers saw the packages to confirm. A continuous count of the different medications was used in this analysis as this reflects both the possible effects of medications themselves and underlying comorbidity.

Depressive mood was assessed through the 20 item Centre for Epidemiological Studies Depression Scale (CES-D)[18]. Cronbach’s alpha for the 20 items in this sample showed good internal consistency (α = 0.87). Self-rated health was assessed with the question: “In general, compared to other people your age, would you say your health is…excellent, very good, good, fair or poor?” Participants were also asked if they were past or current smokers.

### Timed Up-and-Go

The Timed Up-and-Go (TUG) task was measured in both waves. Participants were asked to rise from a chair (seat height 46cm), walk 3m at a normal pace, turn around, walk back to the chair and sit down again [14]. The time taken from the command “Go” to when the participant was sitting with their back resting against the back of the chair was recorded using a stopwatch. TUG was assessed using the same procedure in the home assessment and in wave 2. Researchers in the home assessments in wave 2 sought and conducted the test using a hard-backed chair that matched the health centre chair as closely as possible in height (46cm). TUG is a measure of walking speed, balance and coordination [14]. Higher scores on the TUG task (in seconds) indicate slower walking speed.

### Aging Perceptions Questionnaire (B-APQ)

The short form of the Aging Perceptions Questionnaire (B-APQ) comprises 17 Likert scale items that ask participants to rate their level of agreement with questions about the aging experience and their expectations about aging in the future. The model was based on Leventhal’s self-regulation model of ill health which proposes that the way people think about their own health or illness can be categorised into different themes [19]. The questions are categorised into 5 domains: timeline (e.g. “I am conscious of getting older all of the time”); positive consequences (e.g. “As I get older I continue to grow as a person”); positive control (e.g. “As I get older there is much I can do to maintain my independence”); negative consequences and control (e.g. “Slowing down with age is not something I can control”); and emotional representations (e.g. “I get depressed when I think about getting older”) [20]. Participants rated their level of agreement with each statement as strongly disagree, disagree, neither, agree or strongly agree (range 1–5). The score for each of the five domains was calculated as the average rating of questions within each domain. Cronbach’s alpha showed good internal consistency for each of the domains (Timeline α = 0.75; Positive Control α = 0.85; Negative Control and Consequences α = 0.80; Positive Consequences α = 0.77; Emotional Representations α = 0.75).

### Statistical Analysis

Descriptive statistics were first ascertained for all main variables. TUG time had a skewed distribution which was best corrected with a log transformation. Rather than conducting the analysis on a log-transformed TUG variable, which would have provided output only in the log scale, we conducted a linear regression analysis with a link log function as this allows the predictor to act on the log of the outcome variable while providing marginal mean output in the original scale. This allows for easier interpretation of results.

To test the effect of aging perceptions on change in TUG speed we estimated a second linear regression with follow-up TUG as the outcome, controlling for TUG at baseline as well as all baseline covariates and change in covariates between waves. To better understand the effect of aging perceptions on TUG time we estimated and plotted marginal mean scores of the TUG for five points of the Aging Perceptions scale (1, 2, 3, 4 and 5) which represent the level of agreement with the domain (1 = strongly disagree, 2 = disagree, 3 = neither, 4 = agree, 5 = strongly agree). Marginal means represent the mean in the outcome variable after accounting for the confounding effect of all variables which have been controlled for in the model.

Attrition weights were used due to differences between respondents who participated in both wave 1 and 2 and respondents who participated only in wave 1 on key variables (see Table 1). Attrition weights were calculated as the inverse of the probability that the respondent participated in wave 2 given their participation in wave 1 and their survival up to wave 2. We calculated this using logistic regression in which the dependent variable was whether a respondent had returned in wave 2 or dropped out of the study and the independent variables were multiple measures taken at baseline to try to explain the probability of attrition including measures of baseline mood, cognitive function, physical health, health behaviours and sociodemographic factors. This is multiplied by an initial inverse probability weight reflecting the probability that a member of the Irish population aged 50 years and older participated in the study. This method has previously been reported as an appropriate strategy to minimise bias due to attrition in survey data [21].

#### Sensitivity Analyses

We conducted a number of sensitivity analyses to check the validity of our findings. We had small numbers of missing data in our outcome and covariate variables (<1%) but around 5% of cases for the APQ domains were missing (for numbers see Fig 1). We thus imputed the missing APQ scores using multiple chained imputations. The imputed scores were calculated based on non-missing APQ questions, TUG speed, age, sex and disability.

As a further sensitivity analysis we conducted two further regression analyses which accounted for residual confounding due to measurement error in the baseline TUG measure. Controlling for baseline measures in regression is known to be difficult due to the day-to-day variation within people when taking measures such as walking speed [22]. For the first check we assessed measurement error of the TUG using a second dataset—the Irish data for the Study of Health, Aging and Retirement in Europe (SHARE) data—which has been described in detail elsewhere [23]. This sample included 77 participants who had 2 TUG measures taken with a median lag between assessments of 88 days (IQR 70–104). We then estimated a linear regression using the covariant measurement error (cme) package in Stata 12.1 which estimates the effect of the independent variable on the dependent variable after accounting for the known measurement error of the dependent variable [24]. As there was a 2–6 month gap in measures in the SHARE data we could not control for the possibility that true changes in health accounted for some of the changes in TUG measures rather than purely measurement error. A previous study found that test-retest reliability for the TUG in a sample similar to ours was .97 when the measures were taken within the same day [25]. We thus conducted a second analysis in which we used errors-in-variables regression (EIVREG in Stata V12.1) which is a similar analysis to the covariant measurement error package but which allows input of previous report test-retest reliability coefficients. It has previously been reported to be an appropriate method of analyses of this kind [22].

Finally, we conducted a regression analysis using a change score of TUG time between wave 1 and wave 2 as the dependent variable, both as a sensitivity analysis and in order to calculate marginal mean change in TUG adjusted for all covariates. We conducted a number of further sensitivity analyses including adjustment for type as well as number of chronic conditions and medication. All statistical analyses were conducted using Stata version 12.1.

## Results

Of 5,895 participants who had completed a health assessment and returned in wave 1, 4,944 also completed self-completion questionnaire in wave 1 and a health assessment in wave 2. After exclusion criteria had been implemented and cases with missing data removed the final sample size was 3,913 as illustrated by Fig 1. Mean baseline age of this sample was 62.8 (9.0) years, 54.6% of the sample were female and 35.1% had a third level education. Mean TUG speed in wave 1 was 8.9 (3.0) seconds and 2 years later, in wave 2, was 9.8 (3.9) seconds. Full demographic details are presented in Table 1 including the difference in key variables between participants who returned for wave 2 (N = 4,803), those who died between waves (N = 205) and those who were not followed up due to refusal to participate (N = 809) or inability to contact them (N = 166). Also included is the number of missing data values for each variable.

Table 2 illustrates the bivariate correlations between individual domains of the B-APQ and continuous variables. Table 3 shows the results of the cross-sectional analysis: model 1 indicates the association between the domains of perceptions of aging and walking speed without adjustment for covariates; Model 2 indicates the relationship after adjusting for socio-demographic variables and Model 3 displays the results for the fully adjusted model. All five domains of aging perceptions remained statistically significantly associated with walking speed after full adjustment. The *emotional representations* and *positive control* domains were associated with quicker time in the TUG (Emotional representations: B = -.01 (95% CI:-.02,-.0003); Positive control: B = -.01 (95% CI:-.02,-.001)). The *timeline* (B = .03 (95% CI: .02, .04)), *negative control and consequences* (B = .02 (95% CI: .01, .03)) and, surprisingly, the *positive consequences* (B = .01 (95% CI: .002, .02)) domains were associated with slower TUG times.

**Table 2:** Correlations ^a^ ^b^.

	TC^a^	PC^a^	NCC^a^	PCq^a^	ER^a^	TUG time	Age	Chronic Disease	Medications	Depressed Mood
**TC**	1									
**PC**	-0.07***	1								
**NCC**	0.55***	-0.13***	1							
**PCq**	-0.05***	0.32***	-0.13***	1						
**ER**	0.5***	-0.07***	0.54***	-0.11***	1					
**TUG time**	0.24***	-0.1***	0.26***	-0.05***	0.13***	1				
**Age**	0.23***	-0.09***	0.32***	-0.11***	0.05***	0.42***	1			
**Chronic disease**	0.19***	-0.03*	0.25***	-0.03*	0.18***	0.28***	0.3***	1		
**Medications**	0.22***	-0.05***	0.26***	-0.07***	0.16***	0.31***	0.41***	0.45***	1	
**Depressed Mood**	0.22***	-0.03*	0.24***	-0.08***	0.34***	0.12***	-0.03*	0.23***	0.17***	1
**MMSE**	-0.18***	0.1***	-0.25***	0.06***	-0.12***	-0.29***	-0.32***	-0.12***	-0.18***	-0.08***

Bivariate Correlation Between Domains of the Short-Form Aging Perceptions Questionnaire and all

continuous-covariates.

^a^***p<.001,

**p<.01,

* p<.05.

^b^ TC = Timeline Chronic; PC = Positive Control; NCC = Negative Control and Consequences; PCq = Positive Consequences; ER = Emotional Representations

**Table 3:** Cross-Sectional Relationship^a^ ^b^ ^a^ ^b^.

Timed Up and Go (seconds)	Model 1	Model 2	Model 3
	Coefficient (95% CI)	Coefficient (95% CI)	Coefficient (95% CI)
Timeline	0.05*** (0.04,0.06)	0.04*** (0.03,0.04)	0.03*** (0.02,0.04)
Positive Control	-0.02** (-0.03,-0.01)	-0.01* (-0.02,-0.00)	-0.01** (-0.02,-0.001)
Negative Control and Consequences	0.08*** (0.07,0.09)	0.03*** (0.02,0.04)	0.02*** (0.01,0.03)
Positive Consequences	-0.0004 (-0.01,0.01)	0.01 (-0.0004,0.02)	0.01* (0.002,. 02)
Emotional Representations	-0.03*** (-0.04,-0.02)	-0.005 (-0.01,0.005)	-0.01* (-0.02,-0.0003)
Age		0.01*** (0.01,0.01)	0.01*** (0.01,0.01)
Gender		0.01* (0.001,0.03)	0.01 (-0.01,0.02)
Comparison: Male			
Education:			
Comparison: Primary			
Secondary		-0.03*** (-0.05,-0.02)	-0.005 (-0.02,0.01)
Third/higher		-0.06*** (-0.07,-0.04)	-0.02* (-0.04,-0.00)
Number of chronic diseases			0.01*** (.01,0.02)
Disability			
Comparison: none			
IADL only			0.05* (0.01,0.08)
ADL only			0.09*** (0.06,0.12)
IADL and ADL			0.28*** (0.24,0.32)
No. of medications			0.01*** (0.01,0.01)
Depressed mood			0.001* (0.0001,0.002)
MMSE			-0.01*** (-0.02,-0.01)

Multivariate Regression Model with Coefficients and 95% Confidence Intervals Indicating Relationship between TUG speed and Perceptions of Aging Cross-Sectionally.

^a^ 95% confidence intervals in brackets.

* *p*<0.05,

** *p*<0.01,

*** *p*<0.001.

^b^ADL = Activities of Daily living; IADL = Instrumental Activities of Daily Living; MMSE = Mini-Mental State Examination.

**Fig 2:**
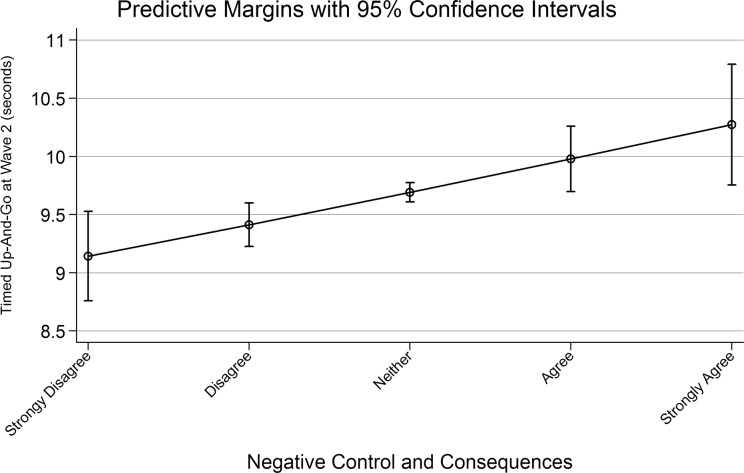
Marginal Mean Timed Up-And-Go. Marginal Mean Timed Up-And-Go in Seconds by Level of Agreement with the Negative Control and Consequences Domain of Aging Perceptions.

Table 4 shows the results of the longitudinal analysis. Model 1 indicates the association between the domains of perceptions of aging and walking speed without adjustment for covariates; Model 2 indicates the relationship after adjusting for socio-demographic variables and Model 3 displays the results for the fully adjusted model including change in covariates between waves. The only domain which was statistically significantly associated with change in walking speed was the *negative control and consequences* domain (B =. 03 (95% CI:. 01,. 05)) which was associated with an increase in time taken to complete the TUG task. Fig 2 displays the marginal mean scores in TUG time in wave 2 by five points along the scale. This represents a difference of approximately 1 second in time taken to complete the task between those who strongly disagreed with the *negative control and consequences* domain and those who strongly agreed with it. We also reran the analysis with change in TUG as the outcome variable which estimated a within-person marginal mean decline of 1.38 seconds (95% CI: .99, 1.76) over two years in those who strongly agreed with the domain versus a decline of .54 seconds (95% CI: .21, .87) for those who did not.

**Table 4:** Longitudinal Relationship^a^ ^b^ ^a^ ^b^.

Wave 2 Timed Up and Go (seconds)	Model 1	Model 2	Model 3
	Coefficient (95% CI)	Coefficient (95% CI)	Coefficient (95% CI)
Timeline	0.02 (-0.004,0.04)	0.01 (-0.01,0.03)	0.003 (-0.02,0.02)
Positive Control	-0.05 (-0.02,0.01)	0.001 (-0.01,0.01)	0.001 (-0.01,0.01)
Negative Control and Consequences	0.09*** (0.06,0.12)	0.06*** (0.03,0.09)	0.03* (0.01,0.05)
Positive Consequences	0.01 (-0.02,0.03)	0.01 (-0.01,0.03)	0.01 (-0.01,0.02)
Emotional Representations	-0.03** (-0.06,-0.01)	-0.01 (-0.04,0.01)	-0.01 (-0.03,0.001)
Timed Up and Go at baseline	0.05*** (0.05,0.05)	0.05*** (0.05,0.05)	0.05*** (0.04,0.05)
Age		0.01*** (0.003,0.01)	0.004*** (0.003,0.01)
Gender		0.04** (0.01,0.06)	0.02* (0.005,0.04)
Comparison: Male			
Education			
Comparison: Primary			
Secondary		-0.02 (-0.06,0.01)	0.01 (-0.02,0.04)
Third/higher		-0.02 (-0.05,0.01)	0.01 (-0.02,0.05)
Depressed Mood (baseline)			0.001 (-0.001,0.003)
Depressed Mood (change)			0.004** (0.001,0.01)
No. of chronic diseases (baseline)			0.01* (0.002,0.03)
No. of chronic diseases (change)			0.02** (0.01,0.04)
Disability			
Comparison: none			
Ongoing disability			0.23*** (0.15,0.36)
Reduced disability			0.10** (0.03,0.14)
New disability			0.12** (0.04,0.19)
Number of reported medications (baseline)			0.005 (-0.002,0.01)
No. of medications (change)			0.005 (-0.0001,0.01)
MMSE (baseline)			-0.02** (-0.02,-0.01)
MMSE (change)			-0.01 (-0.02,0.0001)

Multivariate linear regression analysis indicating the relationship between baseline perceptions of aging and walking speed 2 years later.

^a^ 95% confidence intervals in brackets.

^*^ *p*<0.05,

^**^ *p*<0.01,

^***^ *p*<0.001

^b^ MMSE = Mini Mental State Examination

Finally, we ran a number of sensitivity analyses to check our results. We have included the results of these as supporting information. The multiple imputation model increased our sample size to N = 4461. The results were unchanged—namely that the *negative control and consequences* domain was statistically significantly associated with slower TUG speed at wave 2 (B = .08 (95% CI: .03, .12) (S1 Table). The marginal means indicated a slightly stronger relationship in the imputed model with participants who strongly disagreed with the domain taking an average of 8.27 seconds to complete the TUG task and adults who strongly agreed with the scale taking 11.18 seconds to complete. We have also included the results of the measurement error analyses in the appendix section (S2 and S3 Tables), our findings remained unchanged. We also re-ran all above analyses adjusted for category rather than number of medication (specifically sedative load) and type rather than number of chronic conditions. These changes did not alter our findings and thus we have not presented the data here.

## Discussion

Middle aged and older people who express stronger beliefs in the lack of control and negative consequences of aging have a greater decline in walking speed over 2 years than those who do not. This relationship is not explained by baseline physical function, age, gender, years of education, chronic disease, disability, medication, depressed mood, global cognition or changes in health status over the period. Furthermore, this effect is seen in a relatively healthy cohort free of stroke, dementia and Parkinson’s disease and with an average age of 62.8 years.

One previous study showed that general negative perceptions of aging were associated with decline in objectively measured physical function [13]. Our work expands upon this study to suggest that it is specifically feelings of control and expectations for negative consequences in people’s perceptions of aging which affects decline in walking speed. The saliency of thoughts about aging or the negative emotions associated with aging beliefs do not appear to have an effect. This corroborates previous work by Levy and colleagues who found that the association between self-perceptions of aging and self-reported physical function was partially mediated by perceived control [10]. There is a large body of literature which illustrates the influence of perceived control on health outcomes including cardiovascular disease, depression and functional abilities [26]. Some of the mechanisms thought to explain the relationship between perceived control and health include feelings of decreased self-efficacy, reduced health behaviours and increased stress reactivity [26]. As it appears from our work and Levy et al.’s previous study that control may play an important role in aging perceptions, it is likely that some of the same mechanisms are at play. Here we outline two potential mechanisms which may explain why it is specifically the elements of control beliefs and expectations for negative consequences in aging perceptions that appear to affect decline in objective physical function. We will discuss health behaviours and psychological responses including coping mechanisms.

### Health Behaviours

Previous work has shown that those who blame ‘old age’ as the reason for a chronic condition over any other cause show a decrease in health maintenance behaviours such as having a nutritious diet, participating in exercise, visiting health professionals and getting consistent levels of sleep [27]. Stewart et al. suggested that as aging is uncontrollable, those who blame their condition on old age also see their health as uncontrollable and thus do not act even when the action may be beneficial. Our results indicate that even in a relatively healthy cohort, and after controlling for the presence of chronic conditions, it is the control and expectations for negative consequences aspects of aging perceptions which affect physical function. It is thus possible that these are the same people who blame any health deficits on ‘old age’ and who are less likely to stay physical active, maintain a healthy diet and visit the doctor. Previous work on aging perceptions has shown that adults with positive aging perceptions engage in more exercise, have healthier diets and are more likely to take medications as prescribed than those with negative perceptions regardless of health status [28, 29]. These types of health behaviours are activities which are carried out through the lifespan and not just in older age. It seems unlikely, therefore, that people with negative perceptions of aging make a conscious decision to stop engaging in health maintenance as soon as they reach a certain age. Instead, attentional and confirmatory biases likely reinforce negative perceptions as they age and result in a gradual removal from activities. Psychologically, humans work towards stability in their world view and will give more credence and attention to information which confirms an attitude than to information which disproves it [30]. Adults who have negative perceptions of aging will therefore look for information which confirms that aging is unpleasant. For example, an older adult who believes that severe physical decline is immediately inevitable will have an attentional bias towards the information which confirms this point of view. If they go for a walk and feel more tired than usual after it they will likely attribute this to ‘old age’ rather than considering alternative explanations such as a lack of sleep or a busy week. The negative response to the walk on this occasion may be taken as a sign that walking will always be unpleasant from now on and they will gradually disengage from the activity. As their sedentary behaviour will result in real threats to health, their negative beliefs will be confirmed and the behavioural risks will increase

### Psychological Factors and Coping Mechanisms

The pathway between negative perceptions of aging and declining physical function may not be limited to behavioural explanations but also to psychological responses. We might have expected that the negative emotions elicited by holding negative perceptions of aging would affect walking speed. After all, the effects of depression on walking speed are well documented [31, 32]. Surprisingly, however, although depressed mood was associated with decline in TUG performance, the effect of aging perceptions remained significant after controlling for depression. Furthermore, the emotional representations domain of the B-APQ was not statistically significantly associated with change in TUG in the longitudinal models.

Stress is a potential biological mediator of this effect. Feelings of a lack of control and expectations for negative events are individually associated with increased stress responses both psychologically and physiologically [26, 33]. When a stressful event occurs it activates the hypothalamic-pituitary-adrenal axis which results in spikes in cortisol secretion. Over-activity of this process and resulting sustained high levels of cortisol are damaging to muscle mass [33]. Thus older adults who believe that negative consequences are forthcoming and inevitable, and who believe that they have no control over events, likely experience increased stress and spikes in cortisol secretion which may reduce muscle mass as they age resulting in slower walking speed.

Control beliefs can take two forms: primary control is the belief that behaviours will generate desired effects while secondary control is the belief that one can shape one’s cognitive, emotional or motivational responses to events in the environment [34]. Primary control decreases with age while secondary control increases [34]. Older adults become continuously better at shaping their response to the environment. These coping strategies include selecting and adapting relevant goals in the face of adverse change. For example, an older adult who used to go to the gym daily but who develops a serious illness which prevents them from going could either give up the goal of fitness or could adapt it such that they may aim to be able to walk up a flight of stairs once a day instead. Previous work has shown that older adults with positive perceptions of aging are better at using these goal adaption strategies in the face of serious health events and have higher life satisfaction as a result [7]. Previous work has also shown that older adults primed with negative stereotypes about aging are more likely to dissociate themselves from their peer group [35]. Although this protects self-esteem in the short-term it may also have damaging psychological effects as part of the identity is essentially rejected [6]. Weiss and colleagues suggest that age dissociation may prevent people from reaching important developmental stages in older age such as advance preparation for potential adverse events. This may be related to goal adaptation such that older adults who hold negative perceptions about aging, and who dissociate themselves from their age group as a result, may initially be less likely to prepare themselves for potential adverse events and then less willing to use goal adaptation strategies if adverse events do occur. We thus propose that those adults with positive perceptions of aging are better able to use adaptive strategies to overcome difficulties and are therefore less likely to experience the surge in psychological and physiological stress responses that can affect physical function. It is curious that we did not see a protective effect of the positive control or positive consequences domains on walking speed considering there was a detrimental effect of negative control and consequences. Having examined the individual questions within the domains, however, we suspect that this may be due to the nature of the questions. The positive consequences domain includes statements such as “As I get older I get wiser” which may tap into a different construct than beliefs in the inevitability of physical decline. Similarly, the positive control domain is mostly constructed of questions about the quality of relationships (e.g. “The quality of my relationships with others in later life depends on me”) which, particularly with close family and friends, may not be expected to decline merely due to age.

### Strengths and Limitations

The strength of this study lies in its large, population representative sample and longitudinal data collection. The extent of the measures collected also allowed us to control for a large number of potential confounders which might otherwise explain the association between aging perceptions and physical outcomes, although it is possible that some unmeasured confounding remained. There was some attrition between waves and those who refused at wave 2 had slower walking speed and stronger beliefs in the negative perceptions of aging than those who participated in both waves. However, we attempted to control for the confounding effects of this using attrition weights.

One potential limitation of our study is the missing data values spread across variables. These were small numbers for the majority of variables (for example N = 1 for education and N = 7 for age) but larger for the aging perceptions domains. This is likely due to the nature of the self-completion questionnaire which respondents had to fill in and return to the study themselves. Missing data values were more likely in adults who were older, less educated and less physically able. These, however, are the same variables which the attrition weights attempt to control for and thus some bias due to missing values will have been removed while controlling for attrition although additional confounding may remain. We also imputed the missing data for the aging perceptions domains based on multiple chained imputations but did not find any difference in our results. In fact, the results were slightly stronger in the multiple imputation model suggested that our original model may be conservative. A second limitation is that we only had one measure of aging perceptions at baseline and not at follow-up. This allowed us to assess the independent effect of aging perceptions on TUG speed at wave 2 but not vice versa. It is thus still possible that there could be a reverse causality with an already declining walking speed prior to wave 1 affecting perceptions of aging. We will, however, be taking measures of aging perceptions in future waves of TILDA and thus will be able to update the analysis at a later date in order to assess the change in aging perceptions against change in walking speed.

This study provides evidence for the damaging role of negative perceptions of aging on an objective health outcome and highlights the need for awareness of these potentially modifiable effects. Two previous studies have explored the possibility of changing attitudes towards aging in order to change behaviour (in both cases physical activity). The first study included attribution retraining and physical activity classes in combination. Participants were taught that becoming sedentary is not an inevitable part of aging and that failure to be physically active should be attributed to controllable causes rather than to old age. 3 weeks after the intervention participants were walking more and had increased expectations for aging [36]. The second study was a randomised controlled trial in which adults were categorised into a ‘physical activity only’ or a ‘physical activity with views on aging’ group. The latter group were engaged in sessions which challenged misconceptions about aging and taught participants to recognise and challenge their own automatic negative thoughts. They found that those in the latter group had higher rates of physical activity at follow up than those in the ‘physical activity only’ group [37].

These studies highlight the promising possibility of changing behaviour through changing perceptions of aging. Both studies used a dual model in which general misconceptions of aging were challenged at the same time as changing individual’s attributions about their own experiences of aging. It would be interesting to know which of these components were more effective in changing behaviour and what elements of perceptions of aging they acted upon. For example, it would be interesting to know whether the participants felt more in control of their health, more hopeful about the future or just thought about aging less. Future research could also explore whether the behaviour and attitude change results were long-lasting and what factors determine this. Sarkisian and colleagues, in the 2007 study, found that participants reported improved mood, decreased pain, improved energy and improved sleep following the intervention but they were only followed up for a further 3 weeks. Bodily states and physical perceptions are known to be important predictors of evaluation and attitude formation so it would be interesting to determine whether the improved bodily states of participants changed their experience of how old they felt and resulting in improved self-perceptions of aging in general [36].

In conclusion, slow walking speed, a good marker of overall health among older people, is often seen as a physical, and sometimes inevitable, consequence of old age. Our study indicates that psychological factors, specifically attitudes to aging, could play a significant role in determining the development of this physical symptom.

## Supporting Information

S1 Table Multiple Imputation. Multivariate linear regression analysis in which approximately 5% of the APQ values have been imputed using multiple chained imputations. Indicates the relationship between baseline perceptions of aging and walking speed 2 years later.
(DOCX)

S2 Table Test-Retest Reliability. Multivariate linear regression analysis indicating the relationship between baseline perceptions of aging and walking speed 2 years later adjusted for test-retest reliability.
(DOCX)

S3 Table Measurement Error. Multivariate linear regression analysis indicating the relationship between baseline perceptions of aging and walking speed 2 years later adjusted for measurement error.
(DOCX)

## References

[1] Maurois A. An Art of Living. Ambler, PA: SpiralPress; 2007.

[2] Cesari M, Kritchevsky SB, Penninx BW, Nicklas BJ, Simonsick EM, Newman AB, et al Prognostic value of usual gait speed in well-functioning older people—results from the Health, Aging and Body Composition Study. J Am Geriatr Soc. 2005;53(10):1675–80. . eng. 16181165 10.1111/j.1532-5415.2005.53501.x

[3] White DK, Neogi T, Nevitt MC, Peloquin CE, Zhu Y, Boudreau RM, et al Trajectories of gait speed predict mortality in well-functioning older adults: the Health, Aging and Body Composition study. J Gerontol A Biol Sci Med Sci. 2013 4;68(4):456–64. Pubmed Central PMCID: PMC3593620. eng. 10.1093/gerona/gls197 23051974 PMC3593620

[4] Elbaz A, Sabia S, Brunner E, Shipley M, Marmot M, Kivimaki M, et al Association of walking speed in late midlife with mortality: results from the Whitehall II cohort study. Age (Dordr). 2013 6;35(3):943–52. Pubmed Central PMCID: PMC3636402. eng. 10.1007/s11357-012-9387-9 22361996 PMC3636402

[5] Levy BR. Stereotype Embodiment A Psychosocial Approach to Aging. Curr Dir Psychol Sci. 2009;18(6):332–6. 20802838 10.1111/j.1467-8721.2009.01662.x PMC2927354

[6] Weiss D, Sassenberg K, Freund AM. When feeling different pays off: how older adults can counteract negative age-related information. Psychol Aging. 2013 12;28(4):1140–6. 10.1037/a0033811 23957227

[7] Wurm S, Tomasik MJ, Tesch-Römer C. Serious health events and their impact on changes in subjective health and life satisfaction: The role of age and a positive view on ageing. European Journal of Ageing. 2008;5(2):117–27. 28798566 10.1007/s10433-008-0077-5 PMC5546269

[8] Wurm S, Benyamini Y. Optimism buffers the detrimental effect of negative self-perceptions of ageing on physical and mental health. Psychol Health. 2014;29(7):832–48. eng. 10.1080/08870446.2014.891737 24527737

[9] Kotter-Gruhn D, Kleinspehn-Ammerlahn A, Gerstorf D, Smith J. Self-perceptions of aging predict mortality and change with approaching death: 16-year longitudinal results from the Berlin Aging Study. Psychol Aging. 2009 9;24(3):654–67. 10.1037/a0016510 19739922

[10] Levy BR, Slade MD, Kasl SV. Longitudinal benefit of positive self-perceptions of aging on functional health. J Gerontol B Psychol Sci Soc Sci. 2002 9;57(5):P409–17. . eng. 12198099 10.1093/geronb/57.5.p409

[11] Sargent-Cox KA, Anstey KJ, Luszcz MA. Longitudinal change of self-perceptions of aging and mortality. J Gerontol B Psychol Sci Soc Sci. 2014 3;69(2):168–73. Pubmed Central PMCID: 3968863. 10.1093/geronb/gbt005 23419867 PMC3968863

[12] Wurm S, Tesch-Römer C, Tomasik MJ. Longitudinal findings on aging-related cognitions, control beliefs, and health in later life. The Journals of Gerontology Series B: Psychological Sciences and Social Sciences. 2007;62(3):P156–P64. 17507583 10.1093/geronb/62.3.p156

[13] Sargent-Cox KA, Anstey KJ, Luszcz MA. The relationship between change in self-perceptions of aging and physical functioning in older adults. Psychol Aging. 2012;27(3):750 10.1037/a0027578 22390161

[14] Podsiadlo D, Richardson S. The timed "Up & Go": a test of basic functional mobility for frail elderly persons. J Am Geriatr Soc. 1991 2;39(2):142–8. . eng. 1991946 10.1111/j.1532-5415.1991.tb01616.x

[15] Savva GM, Donoghue OA, Horgan F, O'Regan C, Cronin H, Kenny RA. Using timed up-and-go to identify frail members of the older population. J Gerontol A Biol Sci Med Sci. 2013 4;68(4):441–6. eng. 10.1093/gerona/gls190 22987796

[16] Kearney PM, Cronin H, O'Regan C, Kamiya Y, Savva GM, Whelan B, et al Cohort profile: the Irish Longitudinal Study on Ageing. Int J Epidemiol. 2011 8;40(4):877–84. eng. 10.1093/ije/dyr116 21810894

[17] Folstein MF, Folstein SE, McHugh PR. "Mini-mental state". A practical method for grading the cognitive state of patients for the clinician. J Psychiatr Research. 1975;12(3):189–98. 10.1016/0022-3956(75)90026-6 1202204

[18] Radloff LS. The CES-D scale a self-report depression scale for research in the general population. Appl Psychol Meas. 1977;1(3):385–401.

[19] Barker M, O'Hanlon A, McGee HM, Hickey A, Conroy RM. Cross-sectional validation of the Aging Perceptions Questionnaire: a multidimensional instrument for assessing self-perceptions of aging. BMC Geriatr. 2007;7:9 . Pubmed Central PMCID: PMC1868732. eng. 17462094 10.1186/1471-2318-7-9 PMC1868732

[20] Sexton E, King-Kallimanis BL, Morgan K, McGee H. Development of the Brief Ageing Perceptions Questionnaire (B-APQ): a confirmatory factor analysis approach to item reduction. BMC Geriatr. 2014;14(1):44 . eng. 24716631 10.1186/1471-2318-14-44 PMC4021231

[21] Kessler RC, Little RJ, Groves RM. Advances in strategies for minimizing and adjusting for survey nonresponse. Epidemiol Rev. 1995;17(1):192–204. 8521937 10.1093/oxfordjournals.epirev.a036176

[22] Dugravot A, Guéguen A, Kivimaki M, Vahtera J, Shipley M, Marmot MG, et al Socioeconomic position and cognitive decline using data from two waves: what is the role of the wave 1 cognitive measure? J Epidemiol Community Health. 2009;63(8):675–80. 10.1136/jech.2008.081281 19406741 PMC2789968

[23] Börsch-Supan A, Hank K, Jürges H. A new comprehensive and international view on ageing: introducing the ‘Survey of Health, Ageing and Retirement in Europe’. European Journal of Ageing. 2005;2(4):245–53. 28794739 10.1007/s10433-005-0014-9 PMC5546288

[24] Rabe-Hesketh S, Skrondal A, Pickles A. Maximum likelihood estimation of generalized linear models with covariate measurement error. Stata Journal. 2003;3(4):386–411.

[25] Steffen TM, Hacker TA, Mollinger L. Age-and gender-related test performance in community-dwelling elderly people: Six-Minute Walk Test, Berg Balance Scale, Timed Up & Go Test, and gait speeds. Phys Ther. 2002;82(2):128–37. 11856064 10.1093/ptj/82.2.128

[26] Lachman ME, Neupert SD, Agrigoroaei S. The relevance of control beliefs for health and aging. Handbook of the psychology of aging. 2010:175–90.

[27] Stewart TL, Chipperfield JG, Perry RP, Weiner B. Attributing illness to 'old age:' consequences of a self-directed stereotype for health and mortality. Psychol Health. 2012;27(8):881–97. eng. 10.1080/08870446.2011.630735 22149693

[28] Wurm S, Tomasik MJ, Tesch-Römer C. On the importance of a positive view on ageing for physical exercise among middle-aged and older adults: Cross-sectional and longitudinal findings. Psychology and Health. 2010;25(1):25–42. 10.1080/08870440802311314 20391205

[29] Levy BR, Myers LM. Preventive health behaviors influenced by self-perceptions of aging. Prev Med. 2004 9;39(3):625–9. . 15313104 10.1016/j.ypmed.2004.02.029

[30] Nickerson RS. Confirmation bias: A ubiquitous phenomenon in many guises. Rev Gen Psychol. 1998;2(2):175.

[31] Buchner DM, Cress ME, Esselman PC, Margherita AJ, de Lateur BJ, Campbell AJ, et al Factors associated with changes in gait speed in older adults. J Gerontol A Biol Sci Med Sci. 1996 11;51(6):M297–302. . eng. 8914502 10.1093/gerona/51a.6.m297

[32] Demakakos P, Cooper R, Hamer M, de Oliveira C, Hardy R, Breeze E. The bidirectional association between depressive symptoms and gait speed: evidence from the English Longitudinal Study of Ageing (ELSA). PLoS One. 2013;8(7):e68632 Pubmed Central PMCID: PMC3706406. eng. 10.1371/journal.pone.0068632 23874698 PMC3706406

[33] Peeters G, Van Schoor N, Van Rossum E, Visser M, Lips P. The relationship between cortisol, muscle mass and muscle strength in older persons and the role of genetic variations in the glucocorticoid receptor. Clin Endocrinol (Oxf). 2008;69(4):673–82. 10.1111/j.1365-2265.2008.03212.x 18248637

[34] Sneed JR, Whitbourne SK. Models of the aging self. Journal of Social Issues. 2005;61(2):375–88.

[35] Weiss D, Lang FR. "They" are old but "I" feel younger: age-group dissociation as a self-protective strategy in old age. Psychol Aging. 2012 3;27(1):153–63. 10.1037/a0024887 21988154

[36] Sarkisian CA, Prohaska TR, Davis C, Weiner B. Pilot test of an attribution retraining intervention to raise walking levels in sedentary older adults. J Am Geriatr Soc. 2007;55(11):1842–6. 17979902 10.1111/j.1532-5415.2007.01427.x

[37] Wolff JK, Warner LM, Ziegelmann JP, Wurm S. What do targeting positive views on ageing add to a physical activity intervention in older adults? Results from a randomised controlled trial. Psychol Health. 2014;29(8):915–32. eng. 10.1080/08870446.2014.896464 24559210

