# Supplementary material for: Negative Perceptions of Aging and Decline in Walking Speed: A Self-Fulfilling Prophecy
Source: PLoS One. 2015 Apr 29;10(4):e0123260. doi: 10.1371/journal.pone.0123260 (PMC4414532; doi:10.1371/journal.pone.0123260)
Supplement: S1 Table — Multivariate linear regression analysis in which approximately 5% of the APQ values have been imputed using multiple chained imputations. Indicates the relationship between baseline perceptions of aging and walking speed 2 years later. (DOCX) [file pone.0123260.s001.docx]

**Supporting Information**

**Table S1. Multiple Imputation.^a^**

| **Wave 2 Timed Up and Go (seconds)** | **Model 1** | **Model 2** | **Model 3** |
| --- | --- | --- | --- |
|  | Coefficients (95% CI) | Coefficients (95% CI) | Coefficients (95% CI) |
| Timeline | -.002 (-0.07,0.06) | -0.01 (-0.07,0.05) | -.02 (-0.05,0.01) |
| Positive Control | 0.04 (-0.08,0.15) | 0.04 (-0.08,0.15) | 0.04 (-0.03,0.12) |
| Negative Control and Consequences | 0.16^***^ (0.07,0.25) | 0.11^*^ (0.08,0.05) | 0.08^***^ (0.03,0.12) |
| Positive Consequences | -0.02 (-0.08,0.04) | -.01 (-0.08,0.05) | -.02 (-0.06,0.02) |
| Emotional Representations | -0.003^**^ (-0.07,-0.06) | -0.01 (-0.04,0.01) | -0.01 (-0.03,0.001) |
| Timed Up and Go at baseline | 0.02^***^ (0.01,0.04) | 0.02^***^ (0.01,0.03) | 0.02^***^ (0.01,0.03) |
| Age |  | 0.01^***^ (0.004,0.01) | 0.006^***^ (0.005,0.01) |
| Gender |  |  |  |
| Comparison: Male |  | 0.08^**^ (-0.01,0.16) | 0.05^*^ (0.01,0.08) |
| Education |  |  |  |
| Comparison: Primary |  |  |  |
| Secondary |  | -0.05 (-0.09,0.001) | -0.003 (-0.03,0.03) |
| Third/higher |  | -0.05 (-0.09,-0.02) ^**^ | -0.003 (-0.04,0.03) |
| Depressed Mood (baseline) |  |  | 0.01^**^ (0.004,0.02) |
| Depressed Mood (change) |  |  | 0.01^***^ (0.005,0.02) |
| No. of chronic diseases (baseline) |  |  | 0.01^*^ (-0.00004,0.03) |
| No. of chronic diseases (change) |  |  | 0.003 (-0.02,0.03) |
| Disability |  |  |  |
| Comparison: none |  |  |  |
| Ongoing disability |  |  | -0.004 (-0.27,0.26) |
| Reduced disability |  |  | 0.05^*^ (0.003,0.10) |
| New disability |  |  | 0.24^***^ (0.16,0.33) |
| Number of reported medications (baseline) |  |  | 0.007^*^ (0.001,0.01) |
| No. of medications (change) |  |  | 0.02^**^ (.01,0.04) |
| MMSE (baseline) |  |  | -0.02^**^ (-0.03,-0.01) |
| MMSE (change) |  |  | -0.03^***^ (-0.05,-0.01) |

Multivariate linear regression analysis in which approximately 5% of the APQ values have been imputed using multiple chained imputations. Indicates the relationship between baseline perceptions of aging and walking speed 2 years later.

95% confidence intervals in brackets. ^*^ *p* < 0.05, ^**^ *p* < 0.01, ^***^ *p* < 0.001.
