# Supplementary material for: Negative Perceptions of Aging and Decline in Walking Speed: A Self-Fulfilling Prophecy
Source: PLoS One. 2015 Apr 29;10(4):e0123260. doi: 10.1371/journal.pone.0123260 (PMC4414532; doi:10.1371/journal.pone.0123260)
Supplement: S2 Table — Multivariate linear regression analysis indicating the relationship between baseline perceptions of aging and walking speed 2 years later adjusted for test-retest reliability. (DOCX) [file pone.0123260.s002.docx]

**Supporting Information**

**Table S2. Test-Retest Reliability.**^a^

|  | **Model 1** | **Model 2** | **Model 3** |
| --- | --- | --- | --- |
|  | Coefficients (95% CI) | Coefficients (95% CI) | Coefficients (95% CI) |
| Timeline | -0.04 (-0.17,0.09) | -0.03 (-0.16,0.10) | -0.07 (-0.20,0.05) |
| Positive Control | 0.01 (-0.13,0.16) | 0.017 (-0.13,0.16) | 0.03 (-0.11,0.17) |
| Negative Control and Consequences | 0.42^***^ (0.27,0.57) | 0.38^***^ (0.22,0.54) | 0.20^**^ (0.05,0.36) |
| Positive Consequences | 0.06 (-0.07,0.20) | 0.06 (-0.08,0.20) | 0.03 (-0.10,0.16) |
| Emotional Representations | -0.01 (-0.15,0.13) | 0.001 (-0.14,0.14) | -0.04 (-0.18,0.10) |
| Timed Up and Go at baseline | 1.05^***^ (1.01,1.09) | 1.02^***^ (0.98,1.07) | 0.98^***^ (0.93,1.02) |
| Age |  | 0.02^**^ (0.01,0.03) | 0.01 (-0.00,0.02) |
| Gender |  |  |  |
| Comparison: Male |  | 0.28^**^ (0.10,0.45) | 0.20^*^ (0.02,0.37) |
| Education |  |  |  |
| Comparison: Primary |  |  |  |
| Secondary |  | -0.13 (-0.36,0.11) | 0.10 (-0.13,0.33) |
| Third/higher |  | -0.07 (-0.31,0.17) | 0.18 (-0.07,0.43) |
| Depressed Mood (baseline) |  |  | 0.03^***^ (0.02,0.05) |
| Depressed Mood (change) |  |  | 0.05^***^ (0.03,0.06) |
| No. of chronic diseases (baseline) |  |  | 0.05 (-0.03,0.13) |
| No. of chronic diseases (change) |  |  | 0.09 (-0.01,0.18) |
| Disability |  |  |  |
| Comparison: none |  |  |  |
| Ongoing disability |  |  | 2.16^***^(1.60,2.73) |
| Reduced disability |  |  | 0.28 (-0.08,0.65) |
| New disability |  |  | 1.50^***^ (1.03,1.96) |
| Number of reported medications (baseline) |  |  | 0.0001 (-0.04,0.04) |
| No. of medications (change) |  |  | 0.07^**^ (0.02,0.12) |
| MMSE (baseline) |  |  | -0.14^***^ (-0.21,-0.07) |
| MMSE (change) |  |  | -0.13^***^ (-0.20,-0.07) |

Multivariate linear regression analysis indicating the relationship between baseline perceptions of aging and walking speed 2 years later adjusted for test-retest reliability.

^a^ Test-retest reliability = .97 taken from Steffen et al., 2002. 95% confidence intervals in brackets. ^*^ *p* < 0.05, ^**^ *p* < 0.01, ^***^ *p* < 0.001.
