# Supplementary material for: Negative Perceptions of Aging and Decline in Walking Speed: A Self-Fulfilling Prophecy
Source: PLoS One. 2015 Apr 29;10(4):e0123260. doi: 10.1371/journal.pone.0123260 (PMC4414532; doi:10.1371/journal.pone.0123260)
Supplement: S3 Table — Multivariate linear regression analysis indicating the relationship between baseline perceptions of aging and walking speed 2 years later adjusted for measurement error. (DOCX) [file pone.0123260.s003.docx]

**Supporting Information**

**Table S3. Measurement Error.^a^**

| **Wave 2 TUG** | **Model 1** | **Model 2** | **Model 3** |
| --- | --- | --- | --- |
|  | Coefficients (95% CI) | Coefficients (95% CI) | Coefficients (95% CI) |
| Timeline | -0.003 (-0.01,0.01) | -0.002 (-0.01,0.01) | -0.004 (-0.01,0.004) |
| Positive Control | -0.004 (-0.01,0.01) | -0.004 (-0.01,0.01) | -0.003 (-0.01,0.01) |
| Negative Control and Consequences | 0.03^***^ (0.02,0.04) | 0.03^***^(0.02,0.04) | 0.02^**^ (0.01,0.03) |
| Positive Consequences | 0.007 (-0.00,0.02) | 0.008 (-0.00,0.02) | 0.006 (-0.003,0.01) |
| Emotional Representations | -0.001 (-0.01,0.01) | 0.002 (-0.01,0.01) | -0.002 (-0.01,0.01) |
| Timed Up and Go at baseline | 0.85^***^ (0.81,0.88) | 0.79^***^ (0.75,0.83) | 0.71^***^ (0.66,0.75) |
| Age |  | 0.002^***^ (0.00,0.00) | 0.002^***^ (0.001,0.003) |
| Gender |  |  |  |
| Comparison: Male |  | 0.019^**^(0.01,0.03) | 0.01^*^ (0.003,0.03) |
| Education |  |  |  |
| Comparison: Primary |  |  |  |
| Secondary |  | -0.011(-0.03,0.01) | 0.008 (-0.01,0.02) |
| Third/higher |  | -0.011(-0.03,0.01) | 0.009 (-0.01,0.03) |
| Depressed Mood (baseline) |  |  | 0.002^**^(0.001,0.003) |
| Depressed Mood (change) |  |  | 0.003^***^(0.002,0.004) |
| No. of chronic diseases (baseline) |  |  | 0.001 (-0.01,0.01) |
| No. of chronic diseases (change) |  |  | 0.004 (-0.002,0.01) |
| Disability  Comparison: none |  |  |  |
| Ongoing disability |  |  | 0.18^***^ (0.15, 0.22) |
| Reduced disability |  |  | 0.04^**^ (0.01, 0.06) |
| New disability |  |  | 0.09^**^ (0.06, 0.13) |
| Number of reported medications (baseline) |  |  | 0.004^*^ (0.001,0.01) |
| No. of medications (change) |  |  | 0.004^*^ (0.001,0.01) |
| MMSE |  |  | -0.01^***^ (-0.01, -0.002) |
| MMSE change |  |  | -0.01^**^ (-0.01, -0.002) |

Multivariate linear regression analysis indicating the relationship between baseline perceptions of aging and walking speed 2 years later adjusted for measurement error.

95% confidence intervals in brackets. ^*^ *p* < 0.05, ^**^ *p* < 0.01, ^***^ *p* < 0.001

^a^ Measurement error estimated as the within-person variance in TUG speed in 77 participants from the Study of Health, Aging and Retirement in Europe (within-person variance = .009)
